# A Novel Normalized Cross-Correlation Speckle-Tracking Ultrasound Algorithm for the Evaluation of Diaphragm Deformation

**DOI:** 10.3389/fmed.2021.612933

**Published:** 2021-03-12

**Authors:** Xiong Ye, Zhi Liu, Ying Ma, Ye Song, Lihua Hu, Jianwen Luo, Hui Xiao

**Affiliations:** ^1^School of Clinical Medicine, Shanghai University of Medicine & Health Sciences, Shanghai, China; ^2^National Medical Products Administration (NMPA) Key Laboratory for Respiratory and Anaesthetic Equipment, Shanghai, China; ^3^Department of Biomedical Engineering, School of Medicine, Tsinghua University, Beijing, China; ^4^Department of Ultrasound, Shanghai University of Medicine and Health Sciences Affiliated Zhoupu Hospital, Shanghai, China; ^5^Department of Respiratory and Critical Care Medicine, Shanghai General Hospital, Shanghai Jiaotong University, Shanghai, China

**Keywords:** diaphragm, deformation, ultrasound, speckle tracking, strain

## Abstract

**Objectives:** To develop a two-dimensional normalized cross-correlation (NCC)-based ultrasonic speckle-tracking algorithm for right diaphragm deformation analysis.

**Methods:** Six healthy and eight mechanical ventilation patients were enrolled in this study. Images were acquired by a portable ultrasound system in three sections. DICOM data were processed with NCC to obtain the interframe/cumulative vertical and horizontal displacements, as well as the global strain of the right diaphragm, with continuous tracking and drift correction.

**Results:** The NCC algorithm can track the contraction and relaxation of the right diaphragm by following the respiratory movement continuously. For all three sections, the interframe and accumulated horizontal displacements were both significantly larger than the corresponding vertical displacements (interframe *p* values: 0.031, 0.004, and 0.000; cumulative *p* values: 0.039, 0.001, and <0.0001). For the global strain of the right diaphragm, there was no significant difference between each pair of sections (all *p* > 0.05), regardless of whether the horizontal interval of the initial diaphragm point was 1, 3, 5, or 10 times in the sampling interval.

**Conclusions:** This study developed a novel diaphragm deformation ultrasound imaging method. This method can be used to estimate the diaphragm interframe/accumulated displacement in the horizontal and vertical directions and the global strain on three different imaging planes, and it was found that the strain was not sensitive to the imaging plane.

## Keypoints

- We developed a two-dimensional normalized cross-correlation-based ultrasound speckle-tracking algorithm as a novel method for diaphragm deformation analysis.
- The interpolation of both the radiofrequency signals and cross-correlation function algorithm can be used to track the contraction and relaxation of the right diaphragm with respiratory movement continuously in the horizontal and vertical directions.

## Introduction

The diaphragm is a thin (2–4 mm), movable, dome-shaped, muscular-fibrous structure with a central tendon that separates the thorax from the abdomen and maintains the pressure gradient between those two cavities (1). It is also a vital organ that plays a major role in maintaining ventilation for mammals, as it provides ~80% of the volume capacity for respiration (2). Diaphragm contractility and relaxation occur throughout an individual's life span without resting, not even during sleep; its importance is almost equal to that of the heart, if without normal movement of the diaphragm, life as it is currently known would not be possible (2). The phrenic nerve (PhN), which emerges mainly from the fourth cervical nerve root and partly from the third and fifth nerve roots, maintains the function of the human diaphragm (3). Unilateral PhN injury in most young people without lung pathology is well-tolerated, but in patients with chronic obstructive pulmonary disease, emphysema, or other lung disease may produce symptoms even with mild exertion. Bilateral diaphragmatic paralysis is usually always symptomatic, and the patient may be symptomatic even at rest (4), due to accessory muscles for respiration can compensate only in the short term (5, 6). Protecting the PhN is important because ipsilateral PhN damage significantly reduces the expiratory lung volume, gas exchange, and exercise capacity (7–9). There are various causes of PhN injury, such as cardiac or neck surgery, trauma, pneumonectomy, lung tumors, lung or liver transplantation, metabolic disorders, chronic lung diseases, as well as many systemic diseases, such as multiple sclerosis, that can lead to diaphragm paralysis and ultimately respiratory failure. The use of ice slush is an independent risk factor for PhN dysfunction in patients undergoing cardiac surgery with hypothermic cardiopulmonary bypass (10). Among intensive care unit (ICU) patients, 70% of those with sepsis and multiple organ failure will develop diaphragm dysfunction, which can lead to difficulty in weaning from mechanical ventilation and a prolonged ICU stay (11, 12). Although there are many clinical situations in which the diaphragm can be damaged, the assessment of diaphragm function is often fairly under-recognized (13, 14). The main reason is that there has never been a convenient and accurate method to evaluate the deformation and elastic characteristics of the diaphragm, and there is still a lack of real-time and non-invasive tools for monitoring diaphragm activity at the bedside (15), although such monitoring is sometimes necessary. Currently, Twitch transdiaphragm pressure following magnetic stimulation of the PhN is the gold standard for the non-volitional assessment of diaphragm function (16). Other methods to assess diaphragm function often have little diagnostic value, such as chest X-rays (17), or involve radiation exposure and are difficult to perform at the bedside, as is the case for computed tomography (CT) and magnetic resonance imaging (MRI). Ultrasound is the only non-invasive, radiation-free, portable, and safe diagnostic method with which to directly assess diaphragm motor function in various clinical departments (18–20). Accordingly, many related studies have been published in recent years based on M- and B-mode ultrasound, but the results of each study are quite different. Moreover, M-mode ultrasound provides a one-dimensional measurement of moving tissue along the ultrasound propagation beam. When tissue concurrently moves laterally, this vector cannot be captured (21); consequently, M-mode cannot reflect the true motion of locomotive organs. B-mode ultrasound can only reflect structural information and cannot provide details regarding the elastic characteristics of tissues. Furthermore, both B- and M-mode ultrasound depends on the angle of incidence of the ultrasound beam and requires the operator to have experience.

The ultrasound speckle-tracking imaging technique can provide tissue deformation and more dimensional motion information, such as radial, longitudinal, and circular vectors, which has been successfully used to assess myocardial strain. In 2013, we proposed the concept of diaphragm deformation analysis and used commercial myocardial speckle-tracking software to evaluate diaphragm strain in healthy volunteers (22). Hatam et al. (23) and Orde et al. (24) also used myocardial strain analysis software to evaluate diaphragm strain. All of the above studies were performed using the algorithm designed specifically for myocardium. Currently, in commercial myocardial strain analysis software, analysis of the region of interest is triggered by the R wave of the electrocardiogram, and the analytical process lasts until aortic valve closure. The entire duration of myocardial strain analysis is ~0.02 s, which is much shorter than the duration of inspiration in one respiratory cycle. It is obvious that for the direct analysis of diaphragm deformation using myocardial strain software, the mode of triggering and the duration of the analysis are not consistent with the physiological characteristics of respiration. One potential solution for this limitation is to develop a new specialized algorithm that makes the analytical process cover the entire breathing process or the inspiration phase to fully evaluate the dynamics of the diaphragm (22).

In this study, according to respiratory physiology, we developed a novel speckle-tracking algorithm [i.e., normalized cross-correlation (NCC) algorithm (25)] to evaluate the kinetic characteristics (i.e., displacement and strain) of the right diaphragm first and then carried out a preliminary clinical study in 14 volunteers.

## Methods

### Participants

Six healthy subjects and eight mechanical ventilation patients (a total of 14 participants) were prospectively enrolled in this study. The healthy subjects were examined in the supine position during normal quiet breathing, and the invasive mechanical ventilation patients were lying in bed at a 35-degree angle. The tidal volume of mechanical ventilation was measured with a spirometer for healthy subjects and was obtained from machine settings for mechanical ventilation patients. All of our mechanical ventilation patients were kept in an intubated state using a synchronized intermittent mandatory ventilation model. The ethics committee of the Shanghai University of Medicine & Health Sciences approved this study, and written consent was obtained from all participants.

### Design of Diaphragm Ultrasound Scanning Sections

**Figure 1:**
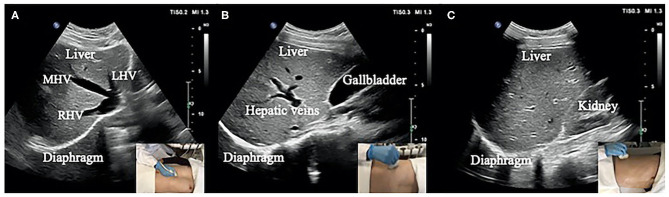
**(A)** Section I: Oblique section of the lower right costal arch through the second hepatic portal with the left hepatic vein (LHV), middle hepatic vein (MHV), and right hepatic vein (RHV) as anatomical markers. **(B)** Section II: Oblique section of the right intercostal passage through the first hepatic portal with the inferior vena cava, hepatic vein, and gallbladder as anatomical markers. **(C)** Section III: Sagittal section of the liver and right kidney with the right kidney and hepatorenal space as anatomical markers.

Movement of the diaphragm in three sections (Figure 1) was assessed by ultrasound using a 3–5-MHz convex-array probe and a LOGIQ V2 ultrasound machine (General Electric Healthcare, Horton, Norway). Twenty-second dynamic digital imaging and communications in medicine (DICOM) images were saved for subsequent ultrasound speckle-tracking imaging analysis. To achieve the desired high-quality boundary of the right diaphragm (26), the dynamic range (e.g., 45–65 dB) was selected based on the clinical application and the patient's condition.

1. Section I: Oblique section of the right costal arch through the second hepatic portal (Figure 1A).
2. Section II: Oblique section of the right intercostal passage through the first hepatic portal (Figure 1B).
3. Section III: Sagittal section of the liver and right kidney (Figure 1C).

### Basic Process of Tracking Diaphragm Deformation

**Figure 2:**
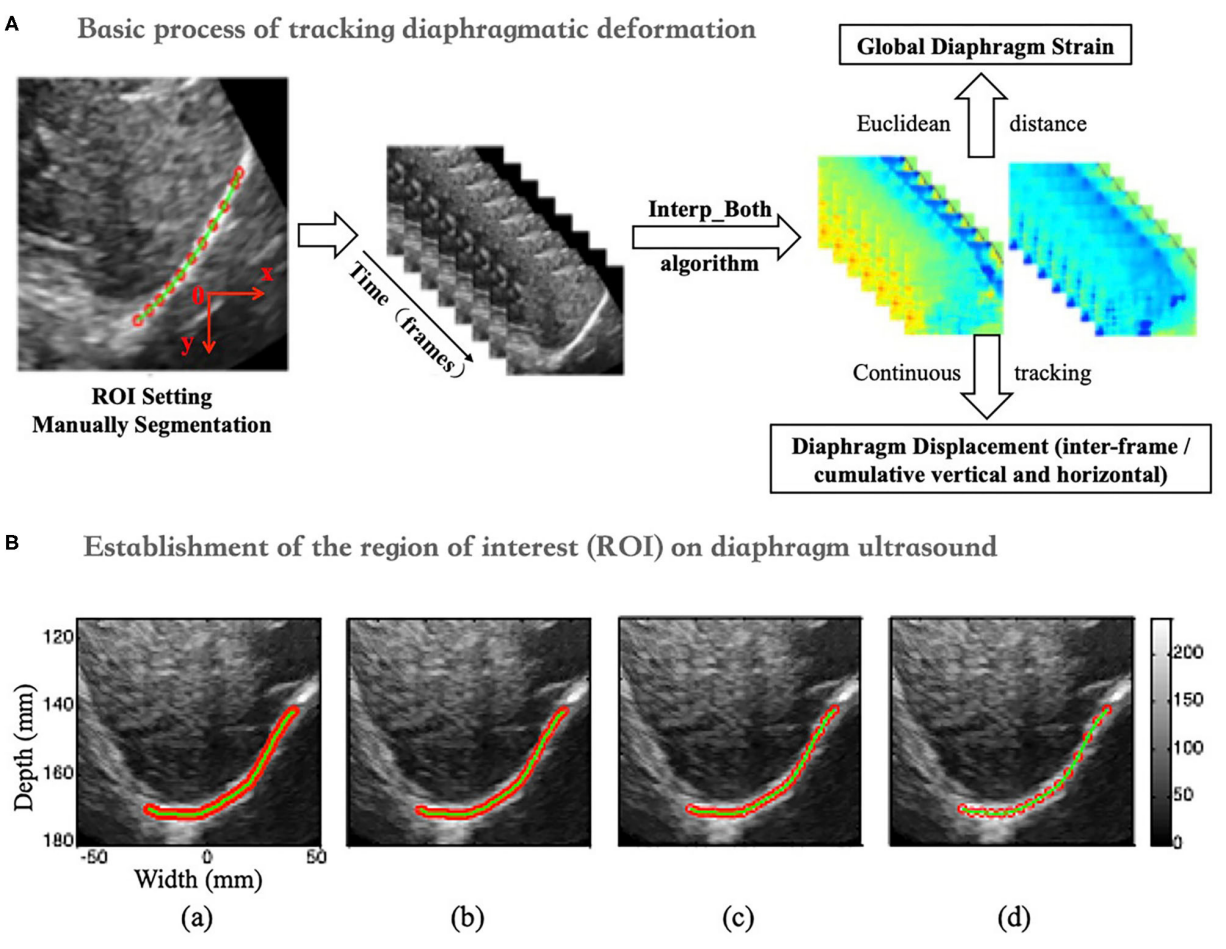
**(A)** Flow chart of diaphragm speckle tracking. **(B)** ROI (initial point) of the diaphragm based on 1- (a), 3- (b), 5- (c), and 10-fold (d) horizontal sampling spacing.

The specific process of diaphragm deformation can be found in Figure 2A. For the normalized cross-correlation-based speckle-tracking method (i.e., lateral interpolation of both the radiofrequency signals and cross-correlation function (Interp_Both), please kindly refer to the study by Liu Z and his colleagues (27).

### Establishment of the Region of Interest (ROI) on Diaphragm Ultrasound

The ROI was set up with four initial points on the diaphragm with 1-, 3-, 5-, and 10-fold (a, b, c, and d) horizontal sampling spacing, as shown in Figure 2B.

### Definition of Diaphragm Displacement and Strain

#### Diaphragm Displacement

**Figure 3:**
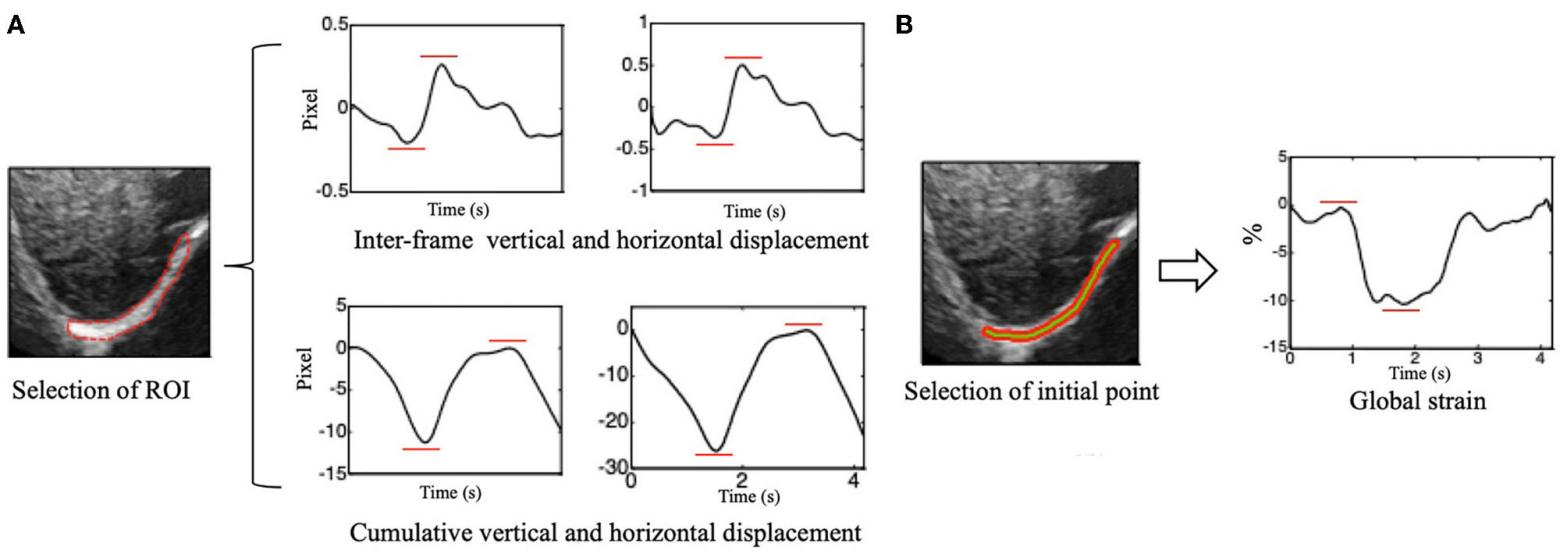
**(A)** Vertical (left side) and horizontal (right side) of inter-frame and cumulative displacement of the right diaphragm in one breathing cycle and schematics of peak-to-peak value extraction. **(B)** Time-dependent global strain curves of the right diaphragm and schematic of peak-to-peak value extraction.

The peak-to-peak value of the overall interframe/cumulative vertical and horizontal displacement was extracted as the displacement of the right diaphragm in one breathing cycle by taking the mean and median displacement of all points in the diaphragm ROI (Figure 3A).

#### Global Diaphragm Strain

The Euclidean distance between each two adjacent points in the ROI was calculated, which can be considered to be approximately equal to the initial length of the diaphragm (i.e., the length of the green segment shown in Figure 3), and it is marked as L0. Continuous tracking of these initial points and calculation of the sum of their distances can yield the length of the diaphragm at a certain time, which is recorded as Lt (Figure 3B). The global strain (GS) of the diaphragm can then be calculated by the following formula (1).

(1) $$\begin{array}{r} \text{GS}=\frac{\text{Lt}-\text{L}0\;}{\text{L}0}\;*\;100\% \end{array}$$

### Normalization of Interframe Displacement

To eliminate the influence of the frame rate on the estimation of the interframe displacement, the interframe displacement was normalized with the following formula (2).

(2) $$\begin{array}{r} \text{D\_}norm=\frac{\text{FR}}{\text{FR }\max}\;*\text{ D} \end{array}$$

D_norm and D denote the interframe displacement after and before normalization, respectively. FR is the acquisition frame rate of the data used to calculate the interframe displacement D, while FRmax refers to the maximum of all data acquisition frame rates.

### Continuous Diaphragm Deformation Tracking Algorithm (Figure 4A)

1. When calculating the interframe displacement, the selected grid points are represented by black dots. The coordinates of the black dots are assumed to be (x, y), while the vertical and horizontal displacements are represented by V1 (U1) and V2 (U2), respectively, which are obtained from the N-1 and N frames and the N and N+1 frames, respectively.
2. Because the diaphragm is moving at all times during breathing, the selected grid point (i.e., black dot) in frame N-1 has moved to a new position (i.e., green dot) in frame N when calculating the displacement, and its coordinates are assumed to be (x', y'); here, x'= x + U1 and y'= y + V1.
3. We can interpolate linearly the vertical and horizontal displacements of the green dots based on the displacements of the black dots in frame N and express them as V2' and U2'.
4. Finally, when frame N-1 changes to frame N, the interframe vertical displacement of the diaphragm changes from V1 to V2' (instead of V2), the interframe horizontal displacement of the diaphragm changes from U1 to U2' (instead of U2), the cumulative vertical displacement of the diaphragm changes from V1 to V1 + V2', and the cumulative horizontal displacement of the diaphragm changes from U1 to U1 + U2'. Similarly, the GS of the diaphragm can be obtained by applying the above process to continuously track the position of the initial points of the diaphragm, followed by calculating the Euclidean distances between the adjacent points, summing the distances, and applying formula (1).

**Figure 4:**
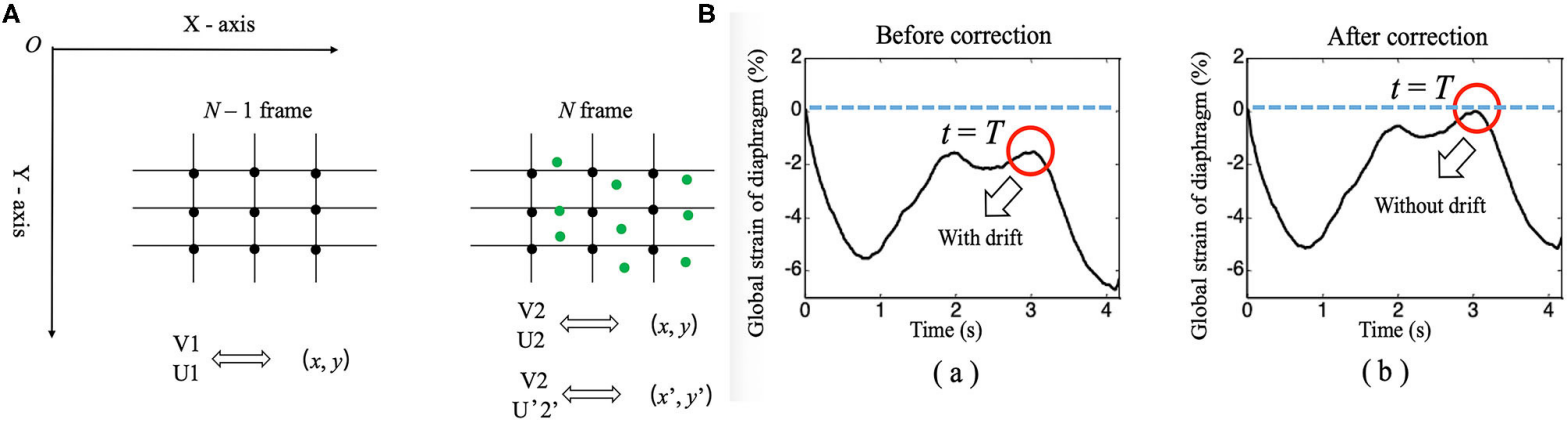
**(A)** Diagram of the continuous tracking algorithm. **(B)** Changes in the global strain curves of the diaphragm correction with time before (a) and after (b).

### Drift Correction Algorithm

An unavoidable problem in continuous tracking is that the displacement or strain curve will drift with the increase in frame number (time), which occurs due to estimation error accumulation in the tracking process (28). Therefore, a drift correction algorithm is used to compensate for the drift in the displacement or strain curve. The process of drift correction is described as follows. First, the movement cycle of the diaphragm with respiration was determined by M-mode ultrasonography, which was assumed to be T. Then, the distribution of the displacement or strain of the diaphragm with time was assumed to be S (t). Displacement or strain begins to accumulate at *t* = 0, and S (T) should return to the original position when *t* = T [i.e., S (T) = 0]. However, due to error accumulation, S (T) is usually not zero. Therefore, to correct the drift of displacement or strain, we adopted formula (3), as follows.

(3) $$\text{S }\_\text{ corr }(\text{t})\text{ = }\{\begin{array}{c} \text{S }\left(\text{t}\right)-\frac{\text{S}(\text{T})}{\text{T}}\;*\text{ t},\text{ 0}\leq \text{ t }\leq \text{T}\\ \text{S }\left(\text{t}\right)-\text{ S }\left(\text{T}\right),\text{ t}\geq \text{T} \end{array}$$

In formula (3), S_corr(t), and S(t) represent the displacement or strain after and before correction, respectively. Figure 4B shows the drift correction effect of this algorithm for the GS of the diaphragm.

### Data Analysis

After correcting the drift, the peak-to-peak values of displacement and strain were extracted to evaluate diaphragm function, and statistical analyses were performed using SPSS 19.0 software (SPSS, Inc., Chicago, IL, USA). All values are shown as the mean ± standard deviation. Descriptive analyses were performed for all investigated variables, and the D'Agostino-Pearson test was used to test for normality. The unpaired *t*-test and non-parametric tests were used to analyze the results of differences in the displacement or strain of the right diaphragm between the two groups, and one-way ANOVA was used for comparing three groups according to data characteristics.

Bartlett's test was used to analyze statistical significance, which was defined as *P* < 0.05.

## Results

### Baseline Characteristics of the Subjects

Diaphragm ultrasound images were acquired from six healthy subjects during quiet spontaneous breathing and eight ICU patients under mechanical ventilation, all of whom were males. The baseline characteristics of all subjects are described in Table 1.

**Table 1:** Baseline characteristics of 14 subjects (mean ± SD).

**Variable**	**Healthy subjects**	**MV patents**	***P***
	**(*N* = 6)**	**(*N* = 8)**	
Age (years)	56 ± 8.2	51 ± 3.6	0.061
BMI (kg/m^2^)	21 ± 5.3	23 ± 2.8	0.084
Tidal volume (ml)	520 ± 17.3	450 ± 38.2	0.102
Ventilation mode	-	SIMV	-

*BMI, body mass index; MV, mechanical ventilation; SIMV, synchronized intermittent mandatory ventilation*.

### Continual Tracking of Diaphragm Movement

**Figure 5:**
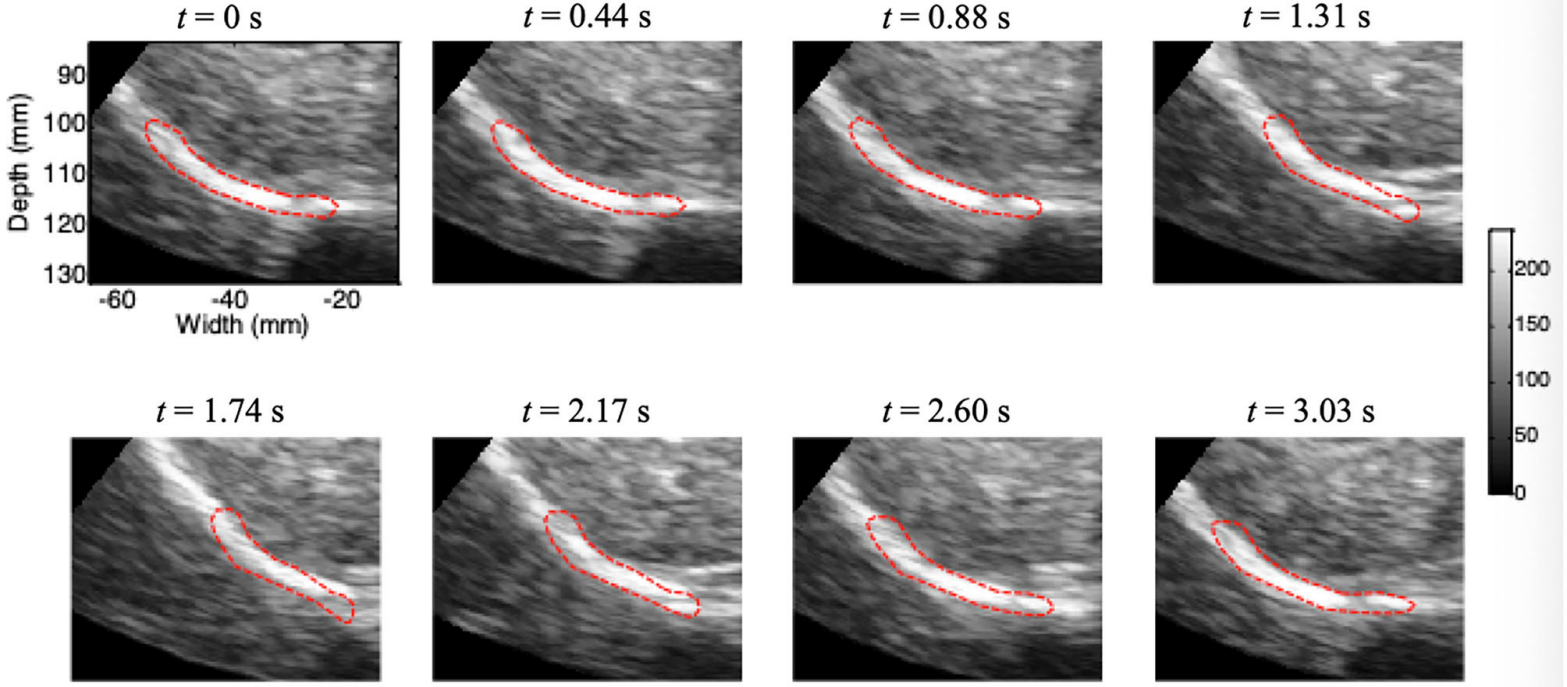
Movement of the diaphragm tracked during one breathing cycle based on the Interp_Both algorithm (red circle, ROI).

Figure 5 shows the results of continuous tracking of the diaphragm ROI during one breathing cycle based on lateral interpolation of both the radiofrequency signals and the cross-correlation function (Interp_Both) algorithm. The ROI of the diaphragm was obtained by manual segmentation at *t* = 0 s. In addition, the ROI of the diaphragm at *t* = 1.31 s was significantly shorter than that at *t* = 0 s, which indicates that the diaphragm was contracting during the inspiratory phase. Then, the diaphragm ROI began to stretch slowly from *t* = 1.31 s to *t* = 2.60 s, which indicated passive relaxation of the diaphragm during the expiratory phase. Most importantly, at the end of the expiratory phase (*t* = 3.03 s), the ROI of the diaphragm essentially returned to the initial position and shape (*t* = 0 s).

### Displacement of the Diaphragm

On ultrasound imaging of the three different sections, there were no significant differences in the peak-to-peak values of the vertical or horizontal displacement and strain of the diaphragm based on the median and mean in the same section, and the *P* values were all >0.05. Therefore, the following results of this study were compared according to the median values.

**Figure 6:**
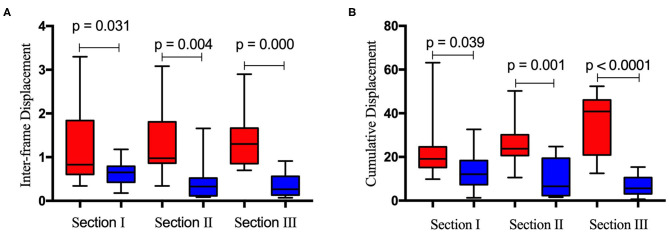
Difference between inter-frame horizontal (red bar) and vertical (blue bar) displacement **(A)** and cumulative horizontal (red bar) and vertical (blue bar) displacement **(B)** of the right diaphragm for the same ultrasound imaging sections.

Figure 6 depicts the horizontal and vertical interframe (Figure 6A) and cumulative (Figure 6B) displacements of the right diaphragm for the same sections. The red and blue boxes represent the horizontal and vertical displacements of sections I, II, and III, respectively. The interframe and cumulative horizontal displacements of sections I, II, and III were significantly greater than the vertical displacements. In terms of significant differences, the *P* values for the differences between the interframe horizontal and vertical displacements of sections I, II, and III were 0.031, 0.004, and 0.000, respectively, while those for the cumulative horizontal and vertical displacements of sections I, II, and III were 0.039, 0.001, and <0.0001, respectively.

### GS of the Diaphragm

Table 2 is based on the differential distribution of the GS of the right diaphragm for the three imaging sections estimated by selecting the initial point of the diaphragm at different sampling intervals. Table 2 shows that when different horizontal intervals were selected to estimate the GS of the diaphragm, the strain values were slightly different. However, after testing the difference in the strain between each pair of sections by one-way ANOVA, it was found that the GS of the diaphragm did not differ significantly by section (*P* >0.05), regardless of whether the horizontal sampling interval of the initial diaphragm point was 1, 3, 5, or 10 (Table 3).

**Table 2:** GS of the right diaphragm determined by selecting the initial point of the ROI based on the horizontal sampling spacing of 1, 3, 5, and 10 for three sections (*N* = 14).

**Horizontal sampling spacing**	**ANOVA summary**		**Bartlett's test**	
	**F (dfn, dfd)**	***P***	**Bartlett's statistic**	***P***
1	0.309 (2, 32)	0.737	10.54	0.005
3	0.793 (2, 32)	0.641	10.81	0.005
5	1.664 (2, 32)	0.205	23.7	<0.0001
10	1.441 (2, 32)	0.252	16.24	0.000

**Table 3:** GS of the right diaphragm determined by selecting the initial point of the ROI based on the horizontal sampling spacing of 5 for the three sections (*N* = 14).

**Section**	**GS (%)**	**95% CI**	***F* (dfn, dfd)**	***P***
	**(Mean ± SD)**			
I	7.89 ± 2.46	6.40 - 9.38		0.218
II	12.9 ± 10.94	5.95 – 19.85	1.602 (2, 31)	
III	10.07 ± 4.00	7.00 – 13.14		

## Discussion

The diaphragm is widely distributed between the thoracic and abdominal regions as the main respiratory muscle pump, including the crural, dorsocostal, midcostal, and ventrocostal regions and the zone of apposition (29). The diaphragm has important physiological functions; thus, evaluating and monitoring changes in its function in the clinic is necessary (30, 31). In ultrasound strain imaging, the displacement and deformation of tissue are estimated using pre- and post-compression imaging data (32). The mechanics of the interaction between the diaphragm and the load are well-understood, but the force-length properties of the diaphragm are non-linear, and algebraic analysis of the interaction is ineffective (33). Ultrasound speckle is granular and textured in appearance due to the backscattered echoes of either randomly or coherently distributed scatterers in tissue. The statistical properties of the received echo speckle signals have been shown to depend on the density and spatial distribution of scatters.

In this study, we developed a new Interp_Both algorithm for ultrasound speckle-tracking imaging of the right diaphragm, and the deformation characteristics of the diaphragm were analyzed successfully by this method. Our previous research showed that Interp_Both with a small interpolation factor (e.g., 3–5) yields the best tradeoff between the estimation accuracy and the time required for computation, thus suggesting such a factor for lateral motion estimation in the case of a low line density (i.e., <2.8 lines/mm) (27). All of the ultrasound data of the right diaphragm that we collected had a low line density. The entire process of active contraction in the inspiratory phase and passive relaxation in the expiratory phase of the right diaphragm could be observed, as shown in Figure 5. The vertical and horizontal movements of the right diaphragm during one respiratory cycle could successfully be tracked continuously. The drift phenomenon could be completely removed from the corrected strain curve by applying the drift correction algorithm. To our knowledge, this is the first study in which a newly designed Interp_Both algorithm based on diaphragm anatomy and respiratory physiology was used to analyze diaphragm deformation. Referencing the standard sections used in liver ultrasonography, we defined three sections of the right diaphragm, which were marked by the three hepatic veins (section I), the inferior vena cava and gallbladder (section II), and the right kidney (section III). According to our newly developed algorithm, the deformation information of these three diaphragm sections was analyzed in 6 normal persons and 8 patients under mechanical ventilation. We used the peak-to-peak values of the instantaneous and cumulative horizontal and vertical displacements and the GS of the right diaphragm as indexes of diaphragm kinetics. The dynamic index of strain reflects the internal characteristics and provides insight into the elastic properties of tissues in either active or passive contraction processes.

This work yielded several interesting findings. First, the instantaneous horizontal displacement was larger than the vertical displacement at both the interframe and cumulative level for the same section, as shown in Figure 6, which indicates that the diaphragm moves horizontally more than vertically in the inspiratory period. Understanding regional diaphragm mechanics and kinematics is very important; however, no studies have shown how the diaphragm moves in different directions during the breathing cycle. Brooke Greybeck and his team studied four dogs and showed that the volume displacement of the diaphragm was heterogeneous and dependent on regional muscle shortening, posture and level of muscle activation (34). Another finding was that the GS of the diaphragm obtained for the three sections did not differ significantly (*P* > 0.05), regardless of whether the horizontal sampling interval of the initial diaphragm point was 1, 3, 5, or 10. This shows that for the diaphragm strain, the section selected has little effect. Furthermore, the initial points of the diaphragm ROI determined with different sampling spacings at different levels have little influence on the final results, which indicates that the selection of imaging sections may not be so strict when calculating the GS of the diaphragm. Although the concept of diaphragm strain in this study is consistent with previous studies (22–24), the results are not comparable due to the completely different algorithm used.

There are some limitations to this study. First, although we have established a new method for diaphragm deformation analysis, the number of samples used for verification was small. Second, the good contrast of the right diaphragm ultrasound images makes it possible to set the ROI, but ultrasound images are probably the most difficult medical images to use for segmentation during image recognition and tracking. Before the ROI set up, we manually segmented the diaphragm images according to the dynamic B- and M-mode ultrasound images, which led to a reduction in the tracking quality. In further research on diaphragm deformation, sensors could be added to display the respiratory waveform on the ultrasound equipment and allow automatic segmentation of the image with the starting point of the inspiratory phase curve as the trigger point for analysis. Because the left diaphragm does not have as nice an acoustic window as the right diaphragm (due to the liver), it is difficult to obtain an ideal image of the left diaphragm. We have not yet attempted to conduct a deformation tracking analysis on the left diaphragm but will in future studies.

## Conclusions

In summary, a novel ultrasound algorithm for deformation (displacement and strain) imaging of the diaphragm based on cross-correlation was developed in our study, and the peak-to-peak values of instantaneous and cumulative displacement and GS were proposed as kinetic indexes of diaphragm function. Differences in the interframe and cumulative vertical and horizontal displacements of the right diaphragm and the GS among three different sections were analyzed. The results show greater horizontal than vertical movement of the diaphragm in each section, and the GS of the diaphragm was not sensitive to the imaging section, which will help reduce the difficulty of choosing the section when performing strain imaging of the diaphragm in the future. In subsequent studies, more clinical data need to be collected to further explore the value and significance of this new technology.

## Data Availability Statement

The original contributions presented in the study are included in the article/supplementary material, further inquiries can be directed to the corresponding authors.

## Ethics Statement

The studies involving human participants were reviewed and approved by Ethics approval was granted by the Human Research Ethics Committee of the Shanghai University of Medicine & Health Sciences, research protocol number 2017/175. The patients/participants provided their written informed consent to participate in this study.

## Author Contributions

XY, HX, ZL, and JL contributed to the design of the study, analysis of the data, and drafting of the manuscript. XY, YM, YS, and LH contributed to obtaining the ultrasound images. All authors critically revised the paper and agreed to be accountable for all aspects of the work.

## Conflict of Interest

The authors declare that the research was conducted in the absence of any commercial or financial relationships that could be construed as a potential conflict of interest.

## Abbreviations

BMI: Body mass index
C T: Computed tomography
DICOM: Digital imaging and communications in medicine
GS: Global strain
MRI: Magnetic resonance imaging
MV: Mechanical ventilation
NCC: Normalized cross-correlation
PhN: Phrenic nerve
ICU: Intensive care unit
Interp_Both: Interpolation of both the radiofrequency signals and cross-correlation function
ROI: Region of interest
SIMV: Synchronized intermittent mandatory ventilation.
